# Reproducibility of deep learning in digital pathology whole slide image analysis

**DOI:** 10.1371/journal.pdig.0000145

**Published:** 2022-12-02

**Authors:** Christina Fell, Mahnaz Mohammadi, David Morrison, Ognjen Arandjelovic, Peter Caie, David Harris-Birtill

**Affiliations:** 1 School of Computer Science, University of St Andrews, St Andrews, United Kingdom; 2 Indica Labs, Albuquerque, New Mexico, United States of America; Yale School of Medicine: Yale University School of Medicine, UNITED STATES

## Abstract

For a method to be widely adopted in medical research or clinical practice, it needs to be reproducible so that clinicians and regulators can have confidence in its use. Machine learning and deep learning have a particular set of challenges around reproducibility. Small differences in the settings or the data used for training a model can lead to large differences in the outcomes of experiments. In this work, three top-performing algorithms from the Camelyon grand challenges are reproduced using only information presented in the associated papers and the results are then compared to those reported. Seemingly minor details were found to be critical to performance and yet their importance is difficult to appreciate until the actual reproduction is attempted. We observed that authors generally describe the key technical aspects of their models well but fail to maintain the same reporting standards when it comes to data preprocessing which is essential to reproducibility. As an important contribution of the present study and its findings, we introduce a reproducibility checklist that tabulates information that needs to be reported in histopathology ML-based work in order to make it reproducible.

## Author summary

For a method to be used a lot in medical research or clinical practice, it needs to be able to be reproduced so that people can trust it. Machine learning and deep learning have some challenges around this. For example, small changes in how a model is trained can lead to significant changes in the results of experiments. This makes it essential that researchers report how they do things in enough detail for the results of their experiments to be reproducible. In this work, we looked at three different algorithms used for digital pathology image analysis. We tried to reproduce them using only the information reported in their papers. We confirmed that even minor details could be essential. Authors often do not report all the details needed to reproduce their work. We also created a checklist of things that need to be reported to help other researchers make their work reproducible.

## 1 Introduction

Digital pathology is a rapidly expanding field of medical imaging. Modern digital scanners allow tissue specimens to be captured at high resolutions (up to 160 nm per pixel), referred to as Whole Slide Images (WSIs). Once samples are available digitally, large displays can replace microscopes, collaborations between clinicians can be done remotely, and the augmentation and automation of the assessment procedure become feasible [1].

Among approaches to automatic WSI assessment, computer vision techniques based on machine learning have been extensively studied [2]. However, for methods to be widely adopted into clinical and research settings, their results must be consistent and reproducible. Machine learning research is undergoing a widely discussed crisis in this regard [3]. Issues such as the inaccessibility of training data, poorly specified methods, and selective reporting of results have led to researchers being unable to recreate the work of others or verify their results [3]. This both slows progress in the field and is highly problematic when moving technologies outside of a research setting.

As a result of these ongoing issues, machine learning publication venues have introduced policies designed to improve reproducibility. For example, in 2019, the Neural Information Processing Systems (NeurIPS) conference introduced a reproducibility program with three components: a code submission policy expecting code for all accepted papers, a reproducibility challenge where members of the community were tasked with reproducing accepted papers, and a checklist of reproducibility best practice [4].

Even as digital pathology becomes increasingly reliant on machine learning techniques, these problems remain under-addressed within the field. In this paper, we report on our attempt to independently reproduce three published algorithms for identifying tumours in whole slide images of breast cancer lymph node tissue. Based on this experience, we identify common weaknesses and omissions within the reporting of these studies, present a checklist for assessing the reproducibility of machine learning methods in digital pathology and score each of the papers against it. Our purpose is to gauge the current state of reproducibility within the field and establish a best practice that can be applied by authors in advance of publication.

## 2 Background

### 2.1 Reproducibility terminology

Although widely encountered in the literature, the term ‘reproducibility’ is often used rather loosely and, even more problematically, is understood in different ways by different authors [5]. A popular choice of terminology is that adopted by the Association for Computing Machinery [6, 7]. It offers the following definitions:

- Repeatability (same team, same experimental setup): the researcher can reliably repeat their own computation.
- Reproducibility (different team, same experimental setup): an independent group can obtain the same results using the authors artefacts.
- Replicability (different team, different experimental setup): an independent group can obtain the same results using different artefacts, developed independently.

In within the context of machine learning, it is useful to consider data and computer code separate experimental artefacts. We want to be able to talk both about results that are that are consistent when a system is tested on new data from the problem domain as well as results that are consistent when the computer code for a system is rewritten (either by the original authors or an independent team). To address these issues, we will use a variation on the above definitions that introduces terms for reproducing studies when the code and/or the data changes. Similar definitions are found in Broman et al. [8] and Raff [9]. The definitions used in this paper are as follows:

- Computational Reproducibility (same code, same data): The ability to reproduce the results of a paper using the same input data and the same code used in the original study.
- Independent Reproducibility (different code, same data): The ability to reproduce the results of a paper using the same input data and code developed independently based on the descriptions in the paper.
- Replicability (different code, different data): The ability to use the techniques described in a paper on new input data of the same type and get the same scientific findings.

### 2.2 Reproducibility best-practices

As machine learning is more widely applied in medical research, the need for reproducibility best-practices has become more urgent. When addressing this, some authors have suggested adopting common programming frameworks and containers [10, 11] while others have suggested structuring medical image analysis challenges in ways that ensure the submitted work is reproducible [12]. To assess the quality of reproducibility specifically within digital pathology, Li et al. [13] independently reproduced a single paper analysing whole slide images, concluding that it’s results are broadly reproducible. Our work contributes to both the understanding the nature of the reproducibility challenge in digital pathology and the establishment of best-practice.

### 2.3 Papers selected to reproduce

When studying reproducibility it is prudent to look at a group of papers that all address the same problem. By doing this, common reproducibility issues can be identified without needing to account for variations in methods introduced by different objectives. Differences in methods are expected or the work would not be novel but at least all authors are attempting the same thing.

**Fig 1:**
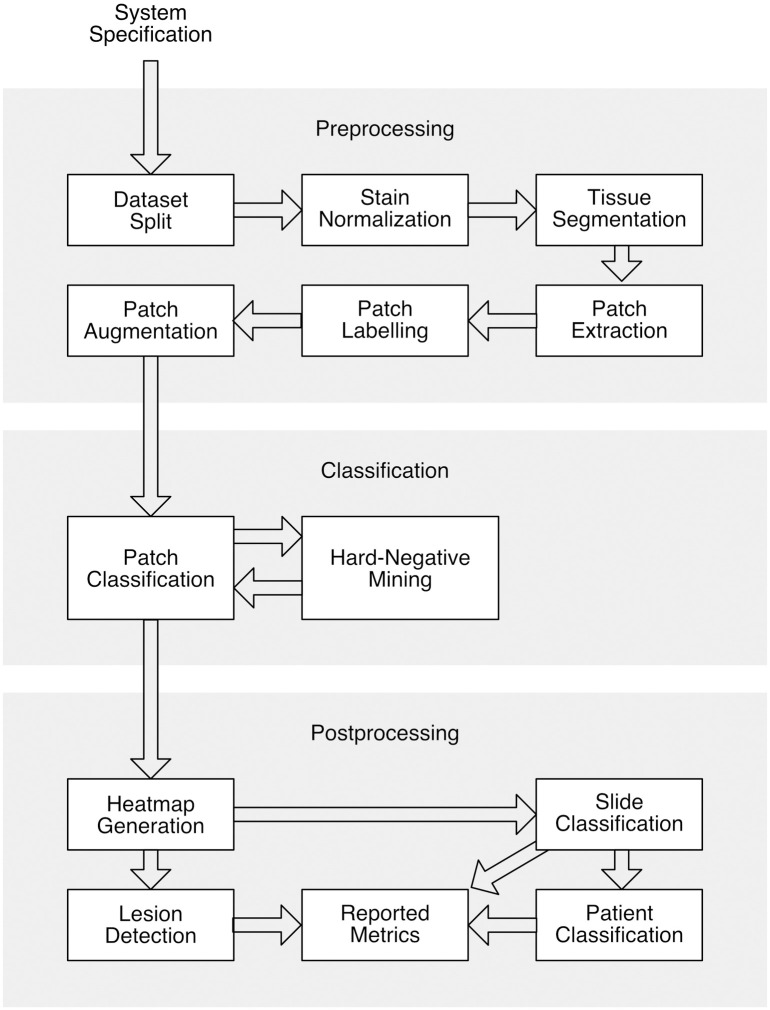
General structure of Camelyon algorithms.

In WSI analysis, such a corpus is available if we collate work done for the Camelyon 16 and 17 challenges [14, 15]. These challenges, organised by Radbound University Medical Center in Nijmegen, The Netherlands, provided WSI datasets extracted from breast lymph nodes and set various tasks for participating researchers. Camelyon 16 focuses on slide level analysis and Camelyon 17 on patient level. Summaries and discussions of the work submitted to these challenges can be found in Bejnordi et al. [16] and Bandi et al. [17]. We found that all the algorithms that were reviewed took a broadly similar technical approach, which we describe in the methods section 3. See Fig 1 for an overview.

It was decided that reproducing three papers would be a sufficient sample size while still remaining practical and that we should select the top performing works as they were likely to be the most influential on future work. Applying these criteria, we selected:

- The winning algorithm submitted to the Camelyon 16 challenge [18].
- A paper by Lee et al. [19] who were the authors of the leading algorithm on the Camelyon 17 leaderboard [20].
- A paper by Liu et al. [21] that had the best reported results on Camelyon 16 when searching in June 2020.

The automation of WSI analysis and diagnosis presents several significant challenges [22]. The WSIs in the Camelyon dataset are approximately 110, 000 × 50, 000 pixels, each utilising around 2GB of storage. In contrast to the images of the ImageNet dataset [23], which are approximately 1.5 GB each in size, several order of magnitude smaller than WSIs, direct application of modern computer vision algorithms considering current hardware computation resources available, is impractical on WSIs. To address this, WSIs are typically downsampled to a more practical size or broken up into smaller patches.

Whole slide images also suffer from a number of unique artefacts. Different labs may use subtly different techniques when extracting, cutting, fixing, staining, and scanning the tissue. These differences can lead to variation in the colour and structure of tissue. In addition, tissue can often take up only a small proportion of the image, potentially leading to wasted computational resources when processing it. Each of our selected papers address these issues in similar but different ways.

For simplicity, from here onward, the algorithms outlined in the three selected papers will be referred to as Wang algorithm [18], Lee algorithm [24] and Liu algorithm [21] respectively.

### 2.4 Checklists

We propose a checklist, see section 4.2, of required information that can be used to improve independent reproducibility in whole slide image analysis. We found that for most papers deep learning techniques were described in sufficient detail, however data pre-processing and post-processing steps were not. Our proposed independent reproducibility checklist covers all parts for the experimental procedure in whole slide image analysis.

In order to approach the creation of the checklist in a principled way, we have attempted to follow guidelines suggested in by Boeing engineer Dan Boorman [25]. These are paraphrased here:

- Pause Point—there should be a clear time to pause and consult the checklist.
- Speedy—it should take less then 60 seconds to complete and have between 5–9 items that must not be missed.
- Supplement to existing knowledge—each item on the list should act as a trigger for the expert, a short and concise reminder of what to do, not a replacement for expertise.
- Field Tested and Updated—the checklist should be be regularly used and updated based on those experiences.

Supplementary to these guidelines, Higgins et. al present a taxonomy of checklists [26], splitting them into:

- Procedural—used for long, complex, or critical tasks where the steps cannot be memorised.
- Preparation—a set of things that must be in-place before an activity is undertaken.
- Problem-solving—a list of questions to ask when trouble shooting something that has gone wrong.
- Prevention—designed to stop critical mistakes from happening.

When developing our checklist we ensured that it followed these guideline and fitted into this taxonomy.

## 3 Methods

When reproducing a paper, some parts of a reported method may be critical to obtaining the same results and confirming the hypothesis and others may be less important. Some reported details may have no effect on the outcome at all. In order to come up with a check list that can help reproducibility in digital pathology, we replicated each paper independently and identified any missing information that would effect replication.

The three selected papers for this experiment follow a similar structure as shown in Fig 1, although individual algorithms may omit or combine some of the steps outlined.

The detailed implementation of each step of the architecture for the selected papers and the results obtained can be found in S1 Text. There are twelve steps, these are:

**System specification**: Giving a technical description of the hardware and software platforms used.

**Dataset split**: Splitting the data into train/valid/test sets used for training, validating and testing the algorithm respectively.

**Stain normalization**: Reducing the colour and intensity variations in stained WSIs from different laboratories.

**Tissue segmentation**: Extracting foreground tissue from the background of the WSI for further processing.

**Patch extraction**: Selecting arbitrary sized patches from WSIs suitable to be fed to CNNs.

**Patch labelling**: Label the extracted patches using the annotations for the WSI.

**Patch augmentation**: Increasing the size of the patch dataset used for training the patch classifier by applying different augmentation techniques.

**Patch classification**: Applying CNNs to the patches of WSIs to classify patches separately.

**Hard negative mining**: Adding or replacing incorrectly classified patches from a previous model to the training dataset and retraining the CNN.

**Heatmap generation**: Converting the output probabilities of the patch classifier to a graphical representation (heatmap) where each pixel value in the heatmap represents probability.

**Slide classification**: Aggregating patch-level results to slide-level results, usually using a classifier trained on the features extracted from heatmaps. This step commonly includes apply various heuristics to the heatmaps to extract features prior to classification.

**Lesion detection**: Using the heatmaps to locate individual lesions within the whole slide image.

**Patient classification**: Combining the results of slide level classification for patients having more than one WSI to get a diagnosis.

**Reported metrics**: Reporting the metrics used to assess the performance of the algorithm and the data on which they were assessed.

In general each step has multiple ways it can be approached. To reproduce each paper exactly, it is necessary to know not only what method has been used, but also the details of multiple parameters that are essential for each step of the algorithm.

Using this architecture to reproduce the outlined papers, allowed us to assess what information was missing, and develop the checklist for reproducibility. The results of this assessment is shown in section 4 along with a comparison of the performance as reported in the papers to that achieved in the reimplementation.

### 3.1 Ethics statement

This work has been approved by the University of St Andrews School of Computer Science Ethics Committee. Approval Code: CS15610.

## 4 Results

Firstly in this section we present the results of our assessment of the reproducibility of each step in the methods of the algorithm from the published papers. Therefore we give the reason for the rating for each step of the method and the decisions made in the reimplementation to fill in missing information.

Secondly in this section we present a comparison of the performance of the published results to those achieved by reimplementation.

### 4.1 Assessment using reproducibility checklist

This section gives the results of using the reproducibility checklist to assess each paper. A summary assessment is provided Table 1 for each step of the general architecture shown in Fig 1. The reasons for these assessments are described in detail in Section 4.1.1 for the paper by Wang et al. [18], in Section 4.1.2 for Lee et al. [19] and in Section 4.1.3 for Liu et al. [21]. These sections also discuss any assumptions that were made to address gaps in the descriptions from the papers or any differences that occurred due to standardising the implementations.

**Table 1:** Assessment of the sufficiency of information provided for reproducibility.

	Wang	Lee	Liu
System specification	✘	✘	✔
Dataset splitting	✘	?	?
Stain normalisation	?	✘	NA
Tissue segmentation	✔	✘	?
Patch extraction	?	?	?
Patch labelling	✘	✘	✔
Patch augmentation	?	NA	✔
Patch classification	✔	?	?
Hard negative mining	✘	?	NA
Heatmap generation	✘	✘	✔
Whole slide classification	?	?	✔
Lesion level classification	✔	NA	✔
Patient level classification	NA	?	NA
Reported Metrics	?	?	✔

In the summary assessment of Table 1: A red background indicates no information was present about that step. A green background colour indicates that all the information required was provided. A yellow colour indicates that some information was provided but a number of assumptions were required in the reimplementation. The steps not applicable for each paper are labelled as “NA”. It should be noted that a step could be assessed as green if only minor information was missing, usually package versions.

What follows is a description of how these assessments were made. We have only included steps where there is something interesting to say about the assessment. Any step that is not present should be assumed to be correctly reported and accurately reimplemented.

#### 4.1.1 Wang algorithm assessment

The winning algorithm submitted to the Camelyon 16 challenge was by Wang et al. [18]. It should be noted that additional details of the algorithm are included in Bejnordi et al. [16] and it’s supplementary details.

**System specification and software—Red**. No information was provided about the hardware or software platform in either Wang et al. [18] or Bejnordi et al. [16]. When reproducing, the hardware and software platform, outlined in the common architecture section of S1 Text, was assumed to be an acceptable choice.

**Dataset splitting—Red**. No information was provided about how or if the slides were split into training and validation sets. For the reproduction, an 80:20 split between training and validation set was applied as it matched the split in the paper by Liu et al. [21] and there was no other information to go on. Splitting was applied at the slide level as outlined in the common architecture section of S1 Text.

**Stain normalisation—Yellow**. Stain normalisation is not mentioned in the paper of Wang et al. [18]. However, in the supplementary details provided by Bejnordi et al. [16], two methods are described: method I, without stain normalisation, and method II, that applies Whole-Slide Image Color Standardizer (WSICS) stain normalisation [27]. WSICS is a template based normalisation technique and the supplementary details did not state what template was used or how it was selected. There is ambiguity in if stain normalisation was applied or not, if it was the technique is clear but there are key parameters missing, therefore it was rated as yellow. As it was unclear which method was reported in Wang et al. [18], stain normalisation was omitted from the reimplementation, this should match the method I reported by Bejnord et al. [27].

**Patch extraction—Yellow**. It is not clear what technique was used for patch extraction, for example if the patches were extracted from random locations, or on an non-intersecting grid. The paper and the supplementary information state different patch sizes: Wang et al. [18] states they were 256x256 and Bejnordi et al. [16] states they were 224 × 224. For the patch sampling strategy, no details are given about if they were balanced across slides or if they were extracted with replacement or not. Due to these pieces of missing information patch extraction was rated as yellow. The reimplementation used a regular grid, patches of size 256 × 256 were used and then cropped down to 224 × 224 as random cropping was stated as one of the augmentations applied to each patch. The assumption was that the cropping from 256 × 256 to 224 × 224 explains the difference in the reported methods.

**Patch labelling—Red**. The description in Wang et al. [18] is unclear as to what area on the patch had to be covered by an annotation polygon for it to be labelled as that class of annotation. Due to the lack of information, when reproducing the patch labelling strategy outlined in the common architecture section of S1 Text, was assumed to be an acceptable choice.

**Patch augmentation—Yellow**. No mention of patch augmentations is given in Wang et al. [18], however in Bejnordi et al. [16] random rotation is stated, but no parameters were given, therefore this was rated as yellow. In the reimplementation a random rotation was selected from 0, 90, 180, or 270 degrees.

**Patch classification—Green**. The details of the software platform, the number of GPUs used and details any parallelism used were missing. Not specifying these details was not a hindrance to reproducing the algorithm, though they may have small variations in the final values reported. As the algorithm described by Wang et al. [18] could still be reproduced this was rated as green.

**Hard negative mining—Red**. It was stated that hard negative mining was carried out, however details of the hard-negative mining were lacking. Therefore, in the reproduction it was assumed that all the false-positive patches were added into the training set and the model was retrained using the weights from the previous training. The previous weights were used in order to take advantage of transfer learning from the existing model and maximise performance. Given the number of false positives that were found, adding them into the training set was deemed not to significantly unbalance the dataset and this seemed like the most straight-forward approach.

**Heatmap generation—Red**. Details of how to generate heatmaps from the probabilities of the patches is not given and so this was rated as red. It is not clear if overlapping patches have been used, if so by how much the patches overlap and how different probabilities in overlapping areas are resolved. Since no more complex details were given, in the reproduction the simplest method that is a regular grid of non overlapping patches, was used.

**Whole slide classification—Yellow**. A random forest algorithm was used to classify the whole slide images based on features extracted from the heatmaps. However, no details of the hyperparameters of the random forest were given or software packages were reported, therefore it was rated as yellow. In the reproduction, the random forest classifier from scikit-learn was trained using it’s default hyperparameters. The defaults were assumed since taking hyperparameters from other studies did not seem sensible as it was assumed any study will have tuned hyperparameters for their own purposes. It is noted in the documentation for the scikit-learn random forest classifier that the defaults can lead to over fitting. When implementing an original model the approach in this situation would be to carry out a hyperparameter search to determine the best settings. When reimplementing from published literature these parameters should be specified so this should not be necessary. With no other information to go from the default parameters have been assumed to be acceptable, if these parameters are critical they should be specified in the published paper.

**Reported Metrics—Yellow**. The paper by Wang et al. [18] reports patch level results but it is not clear if this is on a balanced dataset or all patches from a dataset. It’s also not clear if this accuracy is reported for the test set of the validation set. For slide classification and lesion detection Wang et al. [18] and Bejnordi et al. [16] report slightly different values, we have assumed that the first is reported on valid and second on test. The reported metrics have been classed as yellow due to the lack of clarity on the patch metrics.

#### 4.1.2 Lee algorithm assessment

The leading algorithm on the Camelyon 17 leaderboard [20] in January 2021 is described in [28], the same team has published an alternate version with better results as [19] notably for reproducibility this paper contains more details of the algorithm. This paper was selected to reproduce. The team also provided additional supplementary information as part of the challenge [24].

**System specification and software—Red**. No information was provided about the hardware or software platform in any of the publications [19, 24, 28]. When reproducing the hardware and software platform, outlined in the common architecture section of S1 Text, was assumed to be an acceptable choice.

**Dataset splitting—Yellow**. The information given is the number of patches for each of the train/validation and test sets. There is no information on whether split is done on a patch, slide or patient level and how it is distributed between Camelyon 16 & 17 slides. For reproduction all the slides from Camelyon 16 and and only those from Camelyon 17 with annotations were used for training. Camelyon 16 was split at slide level and Camelyon 17 at patient level to train and validation sets.

**Stain normalisation—Red**. The only information given was that GAN is used for stain normalisation. There was insufficient information to make reasonable assumptions about the technique they have used and so in the reimplementation stain normalisation has not been done.

**Tissue segmentation—Red**. No information has been provided on tissue segmentation. It was assumed tissue segmentation was necessary to help make the implementation more efficient by discarding the parts of the slide that don’t contain any useful information. In the reimplementation the same segmentation method outlined in Liu et al. [21] was applied.

**Patch extraction—Yellow**. It is not clear what technique was used for patch extraction. It was stated that patches were extracted at random without intersection, with no details of how this was implemented. The level at which the patches were extracted was not given and no details were given on how to sample the patches from slides or classes. The patch size and the number of patches for train, validation and test sets were given. The reimplementation sampled patches on a regular grid of size 256 × 256 to ensure no overlapping patches and then randomly sampled from these patches. Finally the sampled patches were cropped down to 240 × 240. As no information was given on what level patches were extracted from, in the reimplementation we extracted patches at level zero as patches extracted at level zero are the highest resolution, this also matched what was done by Wang et al. [18] and Liu et al. [21]. In the reimplementation it was assumed that there was a 50:50 split to balance the classes.

**Patch labelling—Red**. There is no description in Lee et al. [19] as to what area on the patch had to be covered by an annotation polygon for it to be labelled with that annotations class. Due to the lack of information, when reproducing the patch labelling strategy, outlined in the common architecture section of S1 Text, was assumed to be an acceptable choice.

**Patch classification—Yellow**. Not all details needed to carry out patch classification were provided, in particular, the number of epochs to run the training, the batch size and, the loss function were missing. The values chosen for these parameters in the reimplementation were the simplest version of these methods available in the Wang algorithm or the Liu algorithm. The details of the software platform, the number of GPUs used and any parallelism used were also missing, however not specifying these details was not a hindrance to reproducing the algorithm, though they may have small variations in the final values reported.

**Hard negative mining—Yellow**. The paper by Lee et al. [19] does not specify how many patches were added to their initial training dataset by hard negative mining. The initial training dataset for this reimplementation had 45,000 normal patches. When hard negative mining was carried out in the reimplementation 450,000 false positive patches were found. Adding all these patches to the initial training dataset would change both the scale of the training being carried out as well as the balance between normal and tumour patches. It was assumed that if a change this large was found by the authors of the paper [19] then it would have been reported in the paper, perhaps either many fewer false positive patches were found or only a subset of the patches found were added to maintain the scale and balance of the training dataset. It was decided that adding all 450,000 patches found in the reimplementation was not a sensible approach. Therefore the 45,000 of the patches with the highest probabilities found were added to the initial training dataset. To retrain the network with the new training dataset after hard negative mining either the network could be initialised from ImageNet weights or from the weights of the first trained model. Since it was not stated in Lee et al. [19] which one was carried out, the assumption was made to use the weights from the first trained model to take advantage of transfer learning.

**Heatmap generation—Red**. It was stated in Lee et al. [19] that a patch is represented as one pixel in the heatmap, but as there was not enough information on how to extract the patches, it is not clear how the heatmaps are generated. In the reproduction, the simplest method, that is a regular grid of non overlapping patches, was used.

**Whole slide classification—Yellow**. It was stated by Lee et al. [19] that DBScan was used to find clusters of tumor but the parameters used were not stated. A list of 7 features to be extracted from each of the 3 largest tumor clusters found by DBScan was given, this results in 21 features in total. However, it was stated the 24 features were used, and so some information is missing. In the reimplementation the 21 features are used and for DBScan the default parameters are used.

For classification it is stated in Lee et al. [19] that they have used XGBoost but no parameters for this are given. There is further confusion because it is not clear what slides have been used for training and validation and whether the sets used for training and validation at patch level classification is consistent with slide level classification. In the reimplementation, training and validation sets for Camelyon 17 were created at patient level with 62% of the patients in the training set which corresponds to 310 slides in the training. This split remained consistent throughout patch classification, slide classification and patient classification.

Since there are multiple slide for each patient, it is advisable that all the slides for a patient be in the same set due to their similarity. In the paper by Lee et al. [19] there are different splits for patch classification, slide classification and patient level classification which does not seem to maintain the consistency of slides in training and validation sets between patch, slide and patient level classification stages. In the reimplementation it was decided to ensure consistency so that mixing of the train and validation sets could not happen accidentally when moving between different classification stages.

**Patient level detection—Yellow**. The method to go from slide level classification to patient level classification is clearly stated in Lee et al. [19], however as discussed previously the procedure of splitting data is not clear. Therefore we have used the same sets at this stage of classification as we did in patch and slide level classification.

**Reported Metrics—Yellow**. The metrics reported are clearly stated in Lee et al. [19], however as the splits are not clear as stated in 4.1.2, it is not clear what datasets the metrics are reported on.

#### 4.1.3 Liu algorithm assessment

The third paper selected was the one which gave the best results found at the time of the search, in early 2020. This paper by Liu et al. [21] was this final paper selected to reproduce as it had the best reported results on Camelyon 16.

**System specification and software—Green**. The number and type of GPUs used was given by Liu et al. [21], and the software platform used was TensorFlow. In the reimplementation we have used the same number of GPUs but of a different type. It is assumed that GPU architecture is unlikely to have a significant effect on the outcome of the experiment. The reimplementation used PyTorch, there are some differences between PyTorch and TensorFlow but it was expected that these libraries were interchangeable from a methodological point of view.

**Dataset splitting—Yellow**. The method used for allocating slides to training or validation set is not given in Liu et al. [21]. In the reimplementation, the same ratio between the two sets was maintained and slides were sorted randomly into these two sets. A random split, when no other information was provided, is a reasonable way to proceed.

**Tissue segmentation—Yellow**. The level of magnification at which the tissue segmentation was applied was not stated. In the reimplementation, tissue segmentation was carried out at level 5 which was same level in Wang et al. [18]. All other information for tissue segmentation was given and the same was applied in the reimplementation.

**Patch extraction—Yellow**. The size of patches and the level they are extracted are clearly stated in Liu et al. [21], what is not clear is the number of patches used, how they are sampled from the slides and balanced across the classes. The paper presents a patch sampling method designed to avoid bias towards slides with more patches. However, based on the number of patches stated in the appendix and their patch sizes, that would imply they are using all the available patches, meaning that no sampling would be required. In the reimplementation, the number of patches was set as 10,000,000 in the training set with a validation set of 1,250,000. This is in a similar order of magnitude but it’s hard to know the exact numbers from the original system. A similar weighted sampling method was applied when selecting these patches, rather then using all the available patches. Given the uncertainty this may be a source of difference in the results between the original project and the reproduction.

**Patch classification—Yellow**. The description of the patch classifier in Liu et al. [21] was fairly comprehensive, however, the loss function used and the criteria used to determine when training was complete were missing. In our reimplementation cross-entropy loss was used, as this was used with the original GoogLeNet architecture [29]. The model was trained for 15 epochs with early stopping if the validation accuracy didn’t improve for five epochs. The model weights used were those from the epoch with the best accuracy, which was found to be the second epoch.

### 4.2 Checklist for reproducibility of patch-based whole slide image analysis

Given our assessments of the papers, we were able to derive our reproducibility checklist. Placing it within the taxonomy discussed in the background section, it’s clear that it is designed for the prevention of critical errors that can damage the quality of published research. As our checklist covers information that should be present in a research paper, we recommend that the pause point is just before submission of a research paper and associated code but in good time for any omissions to be addressed before the submission deadline. The checklist can also be used procedurally to ensure that experiments are being recorded in the correct level of detail, e.g. all the hyper-parameters for the model are being written down or stored in a database. Our checklist has twelve items, more than the recommended maximum of nine, however this is traded off against being comprehensive for the task at hand. The items are short and designed to act as triggers. As this checklist is a proposal, it seems clear that it will need to be field tested and updated beyond the scope of this paper and we hope that researchers will make use of it in their own work and feedback their experiences to us. We intend to use it in our future work.

In order to make your work independently reproducible, make sure you have reported all the required details of the following:

1. The hardware and software platform the system was trained and tested on.
2. The source of data and how it can be accessed.
3. How the data was split into train, validation, and testing sets.
4. How or if the slides were normalised.
5. How the background and any artefacts were removed from the slides.
6. How patches were extracted from the image and any data augmentation that was applied.
7. How the patches were labelled.
8. How the patch classifier was trained, including technique, architecture, and hyper-parameters.
9. How the slide classifier was trained, including, pre-processing, technique, architecture, and hyper-parameters.
10. How lesion detection was performed.
11. How the patient classifier was trained, including, pre-processing, technique, architecture, and hyper-parameters.
12. All metrics that are relevant to the all the tasks.

### 4.3 Comparison of performance metrics

The following tables show the reported performance metrics from the reproduced papers along side the results from our reimplementations. Results which are not applicable for a particular dataset or task are listed as NA. Results which are missing for a particular task or dataset are listed as -.

The paper by Wang et al. [18] reports patch level results in Table 2 for the GoogLeNet model as having a patch classification accuracy of 98.4%, it is not clear if this is on a balanced dataset or all patches from a dataset. The patch classification results for the reimplementation are on a balanced dataset with an accuracy of 80.5%.

**Table 2:** Comparison of original and reimplementation results of Wang paper.

			Original Paper (Wang)							
Task	Data	Set	Accuracy	Recall	Specificity	Precision	PR-AUC	AUC	Kappa Score	FROC
Patch	Cam16	Valid	0.984	-	-	-	-	NA	NA	NA
Classification		Test	-	-	-	-	-	NA	NA	NA
Slide	Cam16	Valid	-	-	-	-	NA	0.925	NA	NA
Classification		Test	-	-	-	-	NA	0.923	NA	NA
Patient	-	Valid	NA	NA	NA	NA	NA	NA	NA	NA
Classification		Test	NA	NA	NA	NA	NA	NA	NA	NA
Lesion	Cam16	Valid	NA	NA	NA	NA	NA	NA	NA	0.7051
Detection		Test	NA	NA	NA	NA	NA	NA	NA	0.693
			Reimplementation (Wang)							
Patch	Cam16	Valid	0.805	0.715	0.81	0.174	0.43	NA	NA	NA
Classification		Test	0.859	0.731	0.867	0.254	0.579	NA	NA	NA
Slide	Cam16	Valid	0.727	0.782	0.688	0.643	NA	0.891	NA	NA
Classification		Test	0.455	0.45	0.457	0.321	NA	0.493	NA	NA
Patient	NA	Valid	NA	NA	NA	NA	NA	NA	NA	NA
Classification		Test	NA	NA	NA	NA	NA	NA	NA	NA
Lesion	Cam16	Valid	NA	NA	NA	NA	NA	NA	NA	0.456
Detection		Test	NA	NA	NA	NA	NA	NA	NA	0.371

The results reported in Wang et al. [18] give an AUC of 0.925 for the slide classification task. The results reported in c [16] for the HMS and MIT method I are 0.923 with a confidence interval of (0.855–0.977) for the slide classification task.

The results reported in Wang et al. [18] give a score of 0.7051 for the lesion localisation task.

The results reported in Bejnordi et al. [16] for the HMS and MIT method I are 0.693 with a confidence interval of (0.600–0.819) for the lesion identification task.

The authors report the patch level results as “Patch-level classifier shows 0.99 and 0.98 ROC, PR-AUC respectively in both validation and test patches. Optimal threshold in the validation patch set is 0.58, as chosen for the highest F1 score. With this optimal threshold, accuracy, recall, specificity, and precision are 0.99, 0.98, 0.99 and 0.99, respectively, in the validation set”.

Although these results are stated, it’s not clear from the paper if the patches are from Camelyon 16 slides, Camelyon 17 slides, or a combination of the two. It is also not clear if they used all the patches from the slides or sampled in someway, for example to create a balanced subset. The reimplementation results are reported for Camelyon 17 for all patches on the slides.

The slide level results are reported in the paper Lee et al. [24] as “Slide-level accuracy was 0.92 and 0.924 in the validation slides and the entire 500 slides”. It’s unclear where the 500 slides that the paper tested on came from.

Lesion level results are not reported. The slide level results are reported in the paper Lee et al. [24] as “The kappa score for the entire 500 slides is 0.96”. The Camelyon 17 leaderboard [20] gives the patient kappa score as 0.9570.

Patch level results are not reported. For slide level results, the AUC reported in Liu et al. [21] are given as 99.0 on the validation set and 96.7 on the test set. In the reproduction, an AUC of 98.6% was achieved on the Camelyon 16 validation set and 71.8% on the test set.

The lesion level results, reported in Liu et al. [21], gives an FROC of 98.1 on the validation set and 87.3 on the test set for a single Inception V3 net using 40x magnification. In the reproduction, an FROC of 49.9% on the validation set and 3.0% on the test set was achieved.

## 5 Discussion

In this section we discuss two things, firstly the problems that happen when information is missing, and secondly the impact of this missing information has on each papers results. In general it should be noted that it is hard to untangle the effect on the results of any individual missing piece of information without extensive experimentation that controls for many other variables. For example, if information about patch extraction and heatmap generation are both missing, it’s not always clear which one is causing differences from the reported results.

### 5.1 Problems caused by missing information

#### 5.1.1 Mismatched data distributions

The same algorithm trained on different input data can lead to very different output predictions. In particular, differences in the proportion of samples from each class and the data diversity within each class can drastically alter results. The balance and diversity of data is affected by several stages in the pipeline. Such issues affect patch classification, slide classification, and lesion detection.

How the dataset is split into validation and test sets can lead to differences in data distributions between the original experiment and the reimplementation. This can also be the case for how the patches were extracted and balanced between classes. Patch augmentation can also help balance the dataset through increasing the number of samples from a minority class by applying different augmentations such as rotation and flip. Therefore, missing information in any of these can cause the performance of the trained classifier to vary. For example, some samples may be harder to classify than others, if a training set has less of these, it is unlikely to generalise well to a testing set that contains many such samples.

Hard negative mining improves the performance by changing the distribution of the training set, showing the algorithm more of the samples that lead to confusion teaches the classifier to better distinguish between these. Therefore, missing information about the hard negative mining process can affect how the model does or does not generalise to the test set. For example, different ways of adding or replacing samples will lead to differences in the distributions of the training set between the original and reimplementation.

If slides come from different labs, differences in the staining protocols between the labs can lead to differences in the colour distributions of the slides. Stain normalisation compensates for this by transforming each image into the same colour range. Alternatively, patch augmentation can compensate using color augmentations to increase the diversity of the training set. Differences in stain normalisation or colour augmentation due to missing information means that the classifier could be trained on a dataset that is not as representative of the validation and testing sets.

#### 5.1.2 Uncertainty in segmentation and labelling

If areas of the slides are labelled differently in the reimplementation and the original papers this can lead to changes in the classification performance. This can be affected by differences due to missing information in both the patch labelling and tissue segmentation methods.

The amount of tumorous pixels in the patch required for the patch to be labelled as tumor will make it easier or harder to train the patch classifier. For example, if only a few pixels in the patch need to be tumorous to label that patch as tumor, it could be expected that there is more noise and uncertainty in the patch classification step compared to when larger amounts of the pixels in the patch are tumor pixels.

Poor tissue segmentation can cause two problems. Firstly it can exclude tissue that may contain tumor. Secondly including extra background which can introduce noise to the training process. Both of these can affect the patch level classifier results to some extent but their effect on slide and lesion level classification will be larger. Internal holes in the tissue are part of the tissue structure and are of diagnostic importance. Different segmentation methods may include or exclude these regions which can therefore result in different classification accuracies.

#### 5.1.3 Model convergence differences

Missing information can lead to differences in how the model converges for both the patch and slide classification models. The patch and slide classifiers could be overfitted to the data, alternatively they may be underfitted or converge to a local minima.

Here we concentrate on outlining the effects of the patch classification parameters that are missing from the papers, for example if network architecture details were missing that would have a massive effect, but this is not discussed here as these have been well specified. Knowing when to stop the training of the network is a key parameter to replicate performance as it will directly affect over or under fitting of the network. By not specifying other parameters for example, loss functions, optimisers, batch size these are more likely to lead to the network converging to different local minima to the original papers.

Whole slide classification relies on the features extracted from the heatmap to classify each slide. Much like in patch classification, lack of detail about the machine learning technique employed and it’s hyper-parameters can lead to the over or under fitting of the model or the model converging to a different local minima than the one reported.

#### 5.1.4 Dealing with patch classification imperfections

Slide classification and lesion detection could be inferred directly from a perfect patch classification. However, imperfect patch classification needs more complex processing to compensate. Heatmap generation, feature extraction and lesion detection can be carried out to overcome an imperfect patch classification. When any of these steps is not well described differences introduced when reimplementing can reduce the effectiveness. This is now discussed in more detail in the following paragraphs.

The heatmaps are the basic starting blocks for slide, patient and lesion level results and so how they are generated is key to the performance of these. How the heatmaps are generated depends on how the patches are extracted when inference is carried out. Note one set of patches could be used for training the model and then the model applied to a different set to generate the heatmaps. The other key information required is how differences in probabilities between overlapping patches is resolved. For example, averaging over multiple patches at the same location can reduce noise in the heatmaps, extracting patches at different sizes or strides will give different a resolution to the heatmap.

In addition, how the information in the heatmaps is extracted on the performance on the algorithms used for slide and patient classification. Extracting features that are not useful for downstream tasks can add noise the data, conversely, not extracting important features can mean information is lost. Both make the slide classification task more difficult.

Note that weakly-supervised approaches to histopathology slide classification based on Multiple Instance Learning (MIL), such as those evaluated by Sudharshan et al. [30], do not necessarily require heatmaps to be generated. Instead they treat each slide, or part of the slide, as an “bag” of features and train classifiers based on the slide level label. The extraction of these features from the WSI and aggregation of these features depends on the specific method. We are interested in future work to extending our approach and checklist to cover these.

Lesion level detection relies on knowing how to select lesions from the heatmap accurately, identifying true lesions and discarding noise. This is done in two steps: blob detection and blob scoring. There are a number of ways of doing each of these steps and without details of the algorithms used, it can be unclear how to replicate them. Different blob detection algorithms can lead to too many or too few blobs and differences in the scoring methods can make it harder to distinguish between true lesions and false.

#### 5.1.5 Data leakage, maintaining separation between subsets

As discussed in Bussola et. al [31], data leakage is a significant issue in WSI analysis. For example, if the dataset is not partitioned into train and test sets at a patient level then the information can leak from train to test without researchers realising. If the train and test sets contain any of the same data, then the evaluation will be unrepresentative of the models ability to generalise to the target domain. If the train and validation sets contain any of the same data, then the model is likely to overfit. Similar effects can occur when the data is not identical but similar enough to cause issues. For example, two slides that are from the same patient may be very similar and cause overfitting or poor evaluation if used in different splits of the dataset.

To prevent data leakage and maintain separation between subsets, where there are multiple slides per patient, the slides must be split into training, validation, and testing sets at the patient level at the start of the process. In our study, none of the papers included sufficient information to know if the original results were biased by these effects. In our reimplementations, we were careful not to share data between the subsets and it’s unlikely our results were effected by data leakage.

#### 5.1.6 Definition of dataset for results comparison

In order to compare the results, you need to know that you are comparing the same measurements calculated in the same way on the same data. For example, if accuracy is reported, it’s critical to know on which dataset the accuracy was measured, for example validation or test, and how that dataset was created—was it created by sampling all patches within the slide or is it a balanced set created for training. When this information is missing you can end up comparing the same metric on different datasets giving different results.

### 5.2 Impact on reimplementations of missing information

#### 5.2.1 Wang

A comparison of the results reported in [18] and the reimplementation is shown in Table 2. A marked difference can be seen in the patch classification results. In the reimplementation, patch classification accuracy was 15% lower than the value reported in the paper. First of all we do not know if we are comparing the metrics on the same dataset. Secondly dataset splitting, stain normalisation, patch augmentation and hard negative mining all affect the data distribution which can affect the patch classification for the reasons described in Section 5.1.1, and as shown in Table 1 these areas are poorly specified by Wang et al. [18].

In addition, a major difference was seen during slide level classification is shown in Table 2, where the model failed to generalise from the validation set to the test set. The results on the test set were more or less random. As shown in Table 1 there is missing information in patch extraction, heatmap generation and whole slide classification, for the reasons described in Section 5.1.4 these can impact the slide classification results, it may also be due to the differences in data distribution impacting the patch classification. Another possibility is that the random forest classifier used for slide level classification is sensitive to specific hyperparameters and, as these were not stated in the paper, important differences could have arisen.

The lesion level results shown in Table 2 are approximately half as good as those reported in the paper. In lesion level results, the reimplementation sees many more false positives in the predictions. This is an outcome of the lower patch classification accuracy (described above). The lesion identification process is very sensitive to isolated false positive patch predictions, counting each one of these as a separate lesion. As a result, the small difference in patch classification accuracy, increases the average false positive rate for lesions and thus decreases the area under the ROC curve by nearly a half.

#### 5.2.2 Lee

A comparison of the results reported by Lee et al. [19] and the reimplementation is shown in Table 3. It is difficult to figure out on which sets of data the results are reported as shown by the ratings in Table 1. Therefore, we do not know if we are comparing the same results on same datasets in the original paper and the reimplementation.

**Table 3:** Comparison of original and reimplementation results of Lee paper.

			Original Paper (Lee)							
Task	Data	Set	Accuracy	Recall	Specificity	Precision	PR-AUC	AUC	Kappa Score	FROC
Patch	Cam16	Valid	0.99	0.98	0.99	0.99	0.98	NA	NA	NA
Classification	& 17	Test	-	-	-	-	0.98	NA	NA	NA
Slide	Cam17	Valid	0.92	-	-	-	NA	-	NA	NA
Classification		Test	0.924	-	-	-	NA	-	NA	NA
Patient	Cam17	Valid	NA	NA	NA	NA	NA	NA	-	NA
Classification		Test	NA	NA	NA	NA	NA	NA	0.96	NA
Lesion	NA	Valid	NA	NA	NA	NA	NA	NA	NA	NA
Detection		Test	NA	NA	NA	NA	NA	NA	NA	NA
			Reimplementation (Lee)							
Patch	Cam16	Valid	0.94	0.6	0.95	0.15	0.14	NA	NA	NA
Classification	& 17	Test	-	-	-	-	-	NA	NA	NA
Slide	Cam17	Valid	0.5474	-	-	-	NA	-	NA	NA
Classification		Test	-	-	-	-	NA	-	NA	NA
Patient	Cam17	Valid	NA	NA	NA	NA	NA	NA	0.12	NA
Classification		Test	NA	NA	NA	NA	NA	NA	-	NA
Lesion	NA	Valid	NA	NA	NA	NA	NA	NA	NA	NA
Detection		Test	NA	NA	NA	NA	NA	NA	NA	NA

Patch classification is affected by the data distribution as described in Section 5.1.1, the data distribution is affected by the dataset splitting, stain normalisation and hard negative mining and as shown in Table 1 these areas are poorly specified by Lee et al. [19]. Alternatively the missing information on patch labelling and tissue segmentation as shown in Table 1 could affect the patch classification results as described in Section 5.1.2. Finally as shown in Table 1 patch classification parameters are not stated in Lee et al. [19] and differences will affect the results as described in Section 5.1.3. All these reason may contribute to the patch classification precision in the reimplementation being much lower compared to the original.

As shown in Table 3, we were not able to reimplement the slide level results reported by Lee et al. [19]. In addition to the patch level classification problem discussed in the previous paragraph, the heatmap generation, feature extraction and whole slide classification were also lacking in information as shown in Table 1. These affect the slide level results for the reasons described in Section 5.1.4. The missing information therefore impacts on the patient level classification results which should build on top of both the patch and slide level, this is seen when comparing the performance of the patient level classification results of the reimplementation to the original. In addition as shown in Table 1 because the reported metrics are poorly specified, in particular the dataset for the patient level classification, this can affect the patient level results as described in 5.1.5.

#### 5.2.3 Liu

A comparison of the results reported in Liu et al. [21] and the reimplementation is shown in Table 4. As shown in Table 4 the slide classification results reported in Liu et al. [21] are close for the validation set but not for the testing set. This can be due to different distribution of data between validation and testing sets in Liu et al. [21] and the reimplementation. The patch classification parameters are not fully specified in Liu et al. [21] and therefore this can impact the results for the reasons described in Section 5.1.3.

**Table 4:** Comparison of original and reimplementation results of Liu paper.

			Original Paper (Liu)							
Task	Data	Set	Accuracy	Recall	Specificity	Precision	PR-AUC	AUC	Kappa Score	FROC
Patch	Cam16	Valid	-	-	-	-	-	NA	NA	NA
Classification		Test	-	-	-	-	-	NA	NA	NA
Slide	Cam16	Valid	-	-	-	-	NA	0.99	NA	NA
Classification		Test	-	-	-	-	NA	0.967	NA	NA
Patient	NA	Valid	NA	NA	NA	NA	NA	NA	NA	NA
Classification		Test	NA	NA	NA	NA	NA	NA	NA	NA
Lesion	Cam16	Valid	NA	NA	NA	NA	NA	NA	NA	0.981
Detection		Test	NA	NA	NA	NA	NA	NA	NA	0.873
			Reimplementation (Liu)							
Patch	Cam16	Valid	-	-	-	-	-	NA	NA	NA
Classification		Test	-	-	-	-	-	NA	NA	NA
Slide	Cam16	Valid	-	-	-	-	NA	0.986	NA	NA
Classification		Test	-	-	-	-	NA	0.718	NA	NA
Patient	NA	Valid	NA	NA	NA	NA	NA	NA	NA	NA
Classification		Test	NA	NA	NA	NA	NA	NA	NA	NA
Lesion	Cam16	Valid	NA	NA	NA	NA	NA	NA	NA	0.499
Detection		Test	NA	NA	NA	NA	NA	NA	NA	0.03

The lesion level classification results in the reimplementation are poor because lots of noise is detected as lesions which gives lots of false positives. This can be the result of our patch classification not being as good as that of Liu et al. [21]. This is due to the reason described in the previous paragraph.

### 5.3 Limitations and future work

This work contains some important limitations. Independently reproducing work of this kind is time consuming and expensive in terms of computational resources. These have been limiting factors in this study. As we have only reproduced three papers, this limits our ability to draw conclusions about the field in general. However, we believe the pipeline extracted from the papers and checklist can be applied to many of other papers from the Camelyon challenge. It cannot be know what other reproducibilty issues are present in these papers until they are independently reproduced.

Similarly, it is likely that the pipeline and checklist will apply to a wide range of work in the field however the range of their applicability is currently unknown and should be the focus of future work. As noted in the background section, one of the factors that is critical to the success of checklist is that they are tested and updated. This applies both to our pipeline and to our checklist and will be key to their success and to making them more broadly applicable. It is our hope that in the future we can expand the scope of this work significantly by incorporating studies into other published research from a wider range of approaches.

This paper has focused on the problems arising when independently reproducing experiments from the information and details reported in the methods in papers. However, lack of reproducibility can also be due to initially poor experimental design. Rashidi et. al [32] discusses supervised machine learning study design within the context of computational pathology. They recommend reducing model overfitting by introducing cross-validation processes into the training regime. We see this as a different aspect of reproducibility that may be interesting to assess in future work.

## 6 Conclusions

In general, the later stages of the pipelines, such as heatmap generation and patch classification, contained more details and were thus much easier to reproduce. However, the details of the data preparation stages of the pipelines were less well reported. It’s important to understand that although all these papers are using the same datasets (Camelyon16 and Camelyon17), there are many choices to be made between the dataset as distributed and data that is ready for training a CNN. These choices cause the quality of the outcome to vary widely and it is recommended that they should be given equal attention to later stages in the system, such as classification. Without accurate details of data preparation, it is very hard to reproduce work correctly. Reporting the details of data preprocessing steps are as essential as reporting the model and its parameters.

Looking at the results from the above papers, the following trends emerge: issues caused by unclear descriptions of dataset splitting and patch extraction techniques, unclear reporting of results (particularly which subsets of the data the results were reported for), how models were being stopped during training to prevent overfitting and underfitting, the larger the dataset used the better the results were, and the better explained the papers the better the results.

It seems that there is a tension when authoring technical work such as these papers between readability and the completeness required to reproduce the reported work correctly. This is only likely to be solved though the use of extensive supplementary materials, such as detailed appendices and publishing the code. Publishers, editors, and reviews should be aware of these conflicting requirements. Some researchers may also be reticent to publish in the required detail in order to protect commercial interests.

In the process of conducting this analysis it was found to be difficult and almost impossible to reproduce the same results using just the information provided in the published papers. Seemingly minor details can be potentially critical to reproduction and it is difficult to know their importance until trying to reproduce them. A checklist is provided in 4.2 that can help authors to write future papers so that all the necessary steps for reproducibility are included.

## Supporting information

Full details of the implementations and reimplementations of the three algorithms are included in S1 Text. The source code for our reimplementations of the three algorithms is available at the following DOI: 10.5281/zenodo.7014475. We have also factored out much of the functionality of our reimplementations into an open-source Python package called Wsipipe, which is available at the following DOI:10.5281/zenodo.7060584.

S1 Text Supplementary material. General Structure of Camelyon Algorithms: Detailed Description and Implementation Details.
(PDF)
