# Supplementary material for: Reproducibility of deep learning in digital pathology whole slide image analysis
Source: PLOS Digit Health. 2022 Dec 2;1(12):e0000145. doi: 10.1371/journal.pdig.0000145 (PMC9931349; doi:10.1371/journal.pdig.0000145)
Supplement: S1 Text — General Structure of Camelyon Algorithms: Detailed Description and Implementation Details. (PDF) [file pdig.0000145.s001.pdf]

Reproducibility of deep learning in digital pathology whole slide image analysis - supplementary material

Christina Fell[1◑], Mahnaz Mohammadi[1◑], David Morrison[1◑*], Ognjen Arandjelovic[1], Peter Caie[2], David Harris-Birtill[1],

**1** School of Computer Science, University of St Andrews, St Andrews, United Kingdom
**2** Indica Labs, Albuquerque, New Mexico, United States of America

◑These authors contributed equally to this work.
*dm236@st-andrews.ac.uk

# 1 General Structure of Camelyon Algorithms: Detailed Description

General structure of Camelyon algorithms include the following processing steps:

**System Specification** The hardware and the software used should be specified. This includes: a technical description of the hardware used (e.g. CPU, GPU, RAM) and the software platform the project runs on and it's version (e.g. PyTorch, Tensorflow and list of the packages and their versions).

**Dataset Split** The two Camelyon 16 & 17 datasets have distinct training and testing sets, but no uniquely defined validation sets. To be able to validate the performance of the algorithm while training, a validation set must be separated from the training set. This split is done at slide level for Camelyon 16 as there is just one slide per patient and at patient level for Camelyon 17, as there are 5 slides per each patient available. To have the predictions at slide level, often another machine learning step takes place to aggregate the patch level results to slide level predictions. Splitting data at slide level or patient level, prevents data leakage between train and validation sets at patch level training as well as slide level training.

**Stain Normalisation** Staining is used to highlight important features of the tissue as well as to enhance the tissue contrast. Small variations in the multiple steps of the staining process can lead to the variations of the colors in the resulting WSIs. Variations in colors hampers the performance of automatic machine learning based diagnosis. Stain normalisation applies a colour transformation to WSI to normalise all the slides to one standard reference color range. This step is not always applied, colour augmentation may be used instead of or as well as stain normalisation.

**Tissue Segmentation** Approximately 10% of a WSI is tissue and the rest is mostly plain white background. To avoid wasting compute resources on processing the parts of WSI which does not contain useful information, the tissue part is separated from the background. There is a large contrast between the tissue areas and the background. Therefore most approaches apply some form of simple segmentation algorithm to exclude the background to reduce the amount of the image that undergoes further processing. The tissue segmentation typically takes place on a thumbnail from a higher

level of the image pyramid rather than the full resolution whole slide image making it quicker and more efficient.

**Patch Extraction** Due to the large size of WSIs, the limitation of the compute resources and the size of the input that Convolution Neural Networks (CNNs) can handle, the tissue area of WSI should be split to smaller patches suitable to be processed by CNNs, e.g $256 \times 256$ pixels. There are many parameters that determine how this is achieved. It is often done at the lowest level, highest resolution of the image. This also can be done at other or multiple levels. The patches can be created on a regular grid, with a variety of strides or at random from within the tissue area. There are many more patches that do not contain tumour compared to those that are classified as tumour. Therefore most papers in some way reduce the dataset from every possible patch to some specified number or ratio of each class to give more balanced training data.

**Patch Labelling** Extracted patches need to be labelled. At the simplest this is either tumour or normal applied to the whole patch. Alternatively, a mask is created using the annotations provided to show which pixels are tumour and which ones are normal. A patch is then labelled as tumor based on the percentage of its pixels being annotated as tumor. Researchers apply different approaches to how much of the patch needs to be annotated as tumour for the whole patch to be classified as tumour.

**Patch Augmentation** Prior to passing the patches through the classifier augmentation may be carried out. There are a wide variety of techniques applied for example, rotation, mirroring, colour jitter, scaling.

**Patch Classification** The patches are then passed through a supervised CNN based classifier that learns the labels for each patch.

**Hard Negative Mining** Hard negative mining is a technique used to enrich datasets with samples that are difficult to classify in order that downstream classifiers are exposed to harder cases and learn better how to deal with them. It is critical to specify how the dataset in enriched, either though replacing or supplementing existing samples, how the samples were selected, and what percentage of the samples were used.

**Heatmap Generation** Every patch in a whole slide image is then passed though the trained classifier to give a probability of containing tumour for every part of the tissue. These are then reconstructed to give an image where each pixel value represents the probability for that patch.

**Slide Classification** The tumours are then found by setting a threshold or thresholds for the probability. The location and size of these tumours lead to the FROC which is one of the measures used to compare algorithms in Camelyon 16. A variety of measurements of the areas of tumour are then calculated as a new set of features. The feature measurements for each slide are used along with the original classification applied to the slide to train a whole slide classifier, common classification algorithms used are random forests and boosting. Whole slide classification for Camelyon 16 is binary, giving a tumour or normal classification for each slide. This process gives the AUC used to compare the algorithms in Camelyon 16. Whole slide classification for Camelyon 17 is multi-class, giving a none, itc, micro or macro tumor classification for each slide. This process gives us a slide level accuracy used to compare the algorithms in Camelyon 17.

**Lesion Detection** In Camelyon 16 the position of individual lesions are predicted. This used to calculate the Free Receiver Operator Characteristic Curve and score to compare algorithms in Camelyon 16.

**Patient Classification** In Camelyon 17 there are 5 WSIs per patient and the classifications or tumour sizes for all 5 slides can be combined together using rules or by learning another classifier to give a patient level classification.

**Reported Metrics** How a system is assessed is critical to understanding it's usefulness. There are wide range of metrics that can be derived from the results of testing a model. What dataset on which the model is assessed needs to be known and the specific metrics listed. Without both of these it is hard to understand how a reported model performs and thus it's applicability to a problem domain.

# 2 Implementation details as published

## 2.1 Wang algorithm

Details about the winning implementation of the Camelyon 16 challenge come from two places, the paper written by the winning team [1] and the summary paper of the Camelyon 16 challenge [2] in particular the online supplement to this paper. In general the [1] paper gives descriptions of the methods used but does not contain details of parameters, whereas the supplement to [2] is largely a bulleted list with little description but does contain important details of parameters. The winning team presented two variations on their algorithm, the second version called Method II was the winning method.

**System specification and software** No information about the hardware or software platforms used in this experiment was present.

**Dataset splitting** No details are given in the paper by [1] as to how or if the training set of 270 camelyon 16 slides was split into a training or validation set.

**Stain normalisation** The authors do not mention stain normalisation at all in their paper [1]. It is stated in [2] that "(Method II) Staining normalization: Whole-slide image color standardizer (WSICS) [3]" and "(Method I) Staining normalization: None". There is a lack of clarity as to if stain normalization is included or not for method II as it is not mentioned in the paper [1] but is in the [2] paper. It was assumed that if this was a critical detail to the method that this would be included in the paper by the authors.

**Tissue segmentation** Details are given in [1] as "first transfer the original image from the RGB color space to the HSV color space, then the optimal threshold values in each channel are computed using the Otsu algorithm, and the final mask images are generated by combining the masks from H and S channels." According to the supplement [2] to it is carried out at level 5 of the image pyramid.

**Patch extraction** The description in [1] states "We randomly extract millions of small small positive and negative patches", it defines positive and negative as "If the small patch is located in a tumor region, it is a tumor / positive patch and labeled with 1, otherwise, it is a normal / negative patch and labeled with 0.", it does not state how much of the patch has to in the tumour region to be labelled as tumour.

The description in [1] explains states about the patch classification stage that it "uses as input 256x256 pixel patches" Patch classification was evaluated at 40x, 20x and 10x and 40x was found to be the best and this was used for the reported results to Camelyon 16.

The summary information in [2] states the patch size as 224x224 pixels. Furthermore the following useful parameters are included:

- Patches are extracted at level 0 a magnification of 0.24x0.24 $\mu$m.
- the number of training patches per class is 2 million
- patches are uniformly sampled from positive and negative regions.

The two sources mostly supply different information they both supply information on the level at which patches are extracted and the patch size. For the level at which patches are extracted although they use different terminology 40x magnification is the same as level zero or a magnification of 0.24x0.24 mu m so these are in agreement between the two sources. However the two sources list different patch sizes.

It's not clear if the patches were extracted from random locations or on an non-intersecting grid or if they were extracted with replacement or not.

**Patch augmentation** No mention of augmentations is given in [1], however in the [2] the following information is given: "(Method I) Data augmentation: Rotation, random cropping. (Method II) Data augmentation: Rotation, random cropping and addition of color noise"

The software packages and parameters used to perform these augmentations were not detailed.

**Patch classification** A selection of CNNs were explored in [1] but GoogLeNet [4] was found to give the highest patch classification accuracy and was listed as the architecture used in [2].

The following details of the architecture were specified in [2]: "

- Optimization method: Stochastic gradient descent
- Weight initialization: Random sampling from a Gaussian distribution
- Batch size: 32
- Regularization: L 2 regularization (0.0005) and 50% dropout
- Learning rate: 0.01, multiplied by 0.5 every 50,000 iterations
- Activation function: ReLu
- Loss function: Cross-entropy
- Number of training epochs/iterations: 300,000 iteration

"

The software packages used were not detailed.

**Hard negative mining** The authors don't mention if the hard-negative patches were added to the training set or used to replace patches within it, the percentage or number of patches, or if the weights of the pre-hnm classifier were used to initialise the weights of the hnm classifier.

**Heatmap generation** In [2] the heatmap generation is described as "Obtain probability maps from the initial model (the model without hard-negative mining) and the model with hard-negative mining." and in [1] as "After completion of the patch-based classification stage, we generate a tumor probability heatmap for each WSI. On these heatmaps, each pixel contains a value between 0 and 1, indicating the probability that the pixel contains tumor.".

**Whole slide classification** The detail included in [1] is that "we extract 28 geometrical and morphological features from each heatmap, including the percentage of tumor region over the whole tissue region, the area ratio between tumor region and the minimum surrounding convex region, the average prediction values, and the longest axis of the tumor region"

The whole slide classification is described in [2] as follows:

"There are two types of features global and local. The following global features are calculated based on thresholds of 0.5, 0.6, 0.7, 0.8 and 0.9 being applied to the heatmaps.

- ratio between metastatic region and tissue area
- sum of all cancer probabilities in metastatic areas

The local features are calculated based on a threshold of 0.5 being applied to the heatmaps. The two largest connected regions areas found in the binary image and the following [2] states 9 features calculated per region but actually 10 are listed in the paper.

- area of region
- the eccentricity of an ellipse that has the same second moments as the region
- the ratio of the area to the area of the bounding box
- the bounding box area
- the major axis length of the ellipse with the same second moments of area as the region
- the max/mean/min intensity within the region
- the aspect ratio of the bounding box
- the ratio of the area of the region to the area of the convex area

" The classification process is described in [1] as follows "We compute these features over tumor probability heatmaps across all training cases, and we build a random forest classifier to discriminate the WSIs with metastases from the negative WSIs."

The software package and version information was missing. the parameters used to train the random forest classifier were not provided.

**Lesion extraction** For the lesion level task the details given in [1] are as follows "we first train a deep model (D-I) using our initial training dataset described above. We then train a second deep model (D-II) with a training set that is enriched for tumor-adjacent negative regions. This model (D-II) produces fewer false positives than D-I but has reduced sensitivity. In our framework, we first threshold the heatmap produced from D-I at 0.90, which creates a binary heatmap. We then identify connected components within the tumor binary mask, and we use the central point as the tumor location for each connected component. To estimate the probability of tumor at each of

these (x, y) locations, we take the average of the tumor probability predictions generated by D-I and D-II across each connected component.” The software package and version information was missing. The detail given in [2]. ”

- Obtain probability maps from the initial model (the model without hard-negative mining) and the model with hard-negative mining.
- Threshold the probability map of the initial model at 0.9.
- Extract connected components.
- Take the center point of each connected component as the lesion location.
- The lesion probability score is calculated as the sum the values in that region in both probability maps.
- (Method II only) Each lesion score is additionally weighted by the slide-based score (obtained from the whole-slide image classification task).

”

**Patient level stage classification** The paper uses only Camelyon 16 so does not report patient level classification.

## 2.2 Lee algorithm

In [5], a machine learning process for predicting slide-level cancer metastasis is described. The details of this implementation is summarised as following:

**System specification and software** The authors of the paper [6] have not mentioned the specifications of the system the have run the experiments on. The software framework version and the packages and libraries used for this purpose also have not been reported in the paper.

**Dataset splitting** The authors of the [5] paper state ”The data set for training patch classifier comes from both Camelyon 16 and Camelyon 17 datasets”. In addition it was stated that ”This results in total number of 95149, 58000, and 48000 patches in training, validation and test sets respectively”. They have not mentioned if they are using all the slides in both datasets at this stage. Some slides in Camelyon 17 dataset don’t have any annotations and ideally can not be included for training or evaluation at patch level.

**Stain normalisation** Authors of paper [6] state that ”Assuming that the ’16 dataset contains two different medical centers, total seven different stain styles are included.” The authors state ”we normalise the colour with Generative Adversarial Network (GAN)”, no further details are given on the type of GAN and the parameters used for this purpose.

**Tissue segmentation** The authors make no mention of tissue segmentation.

**Patch extraction** The authors state ”patches of 240 $\times$ 240 pixels are extracted randomly without intersection from both cancerous and non-cancerous regions of slides”. It does not state at what magnification or level of the pyramid patches were extracted. The authors do not mention how much of the patch has to be in a cancerous region to be classed as cancerous. Number of tumour/normal patches extracted has not been reported. What is reported is the total number of patches extracted from the slides used for training.

**Patch augmentation** No augmentations were mentioned by the authors.

**Patch classification** The patch classifier is described by the authors as: "Patch classifier is trained by DenseNet-121 model pretrained with 1000-class ImageNet dataset.". The net is adapted as described "a fully-connected layer from 1000 to 2 is added at the end of the original version.". The following information is given about key hyperparameters, "Initial learning rate is 0.1 and reduced by, one tenth per 10 epochs, and the optimiser is SGD with 1e-4 decay.". The authors do not report the patch classifier network they have used is from which package and what version of the package is used. The is no report of the number of epoch the network has been trained for, the batch size, if any early stopping is done or not and if any type of data or model parallelism is done while training the model. They have also not reported if the training has been done using GPUs and if yes what number of GPUs have been used.

**Hard negative mining** The authors state "An additional hard negative mining step is then carried out, this is described by the authors as "Additional normal patches are chosen from the heatmap regions that disagree the most with the reference annotation. Finally, the same patch classifier is trained again with the dataset with the additionally extracted normal types." It is not reported how the initialisation of the weights has been done to re-train the model at this stage.

**Heatmap generation** The description from the authors of how the heatmap was created is "By using the patch classifier, each whole slide image is transformed to a heatmap which considers a 240-by-240 pixel patch as a single pixel".

**Whole Slide Classification** The description given by the authors of how to convert the heatmap into features is "To classify slide-level metastases, morphological features from heatmap are extracted by DBSCAN algorithm. Per each of the three largest clusters within a slide, features such as the major axis, minor axis, area, density, mean probability, max probability, and min probability are extracted." The parameters of DBScan and the version of the package used for feature extraction are not reported. There is no proper description of the features in the paper. According to the paper the morphological features extracted from each of the three largest clusters within a slide, features are the major axis, minor axis, area, density, mean probability, max probability, and min probability are extracted." According to the paper total of 24 features have been extracted for each slide, this needs 8 features to be extracted from each of the three largest clusters within a slide, but only 7 features have been mentioned by the authors.

The description given by the authors is "Slides with 24 features are trained by XGBoost." Furthermore they specify the training set used for this classifier as "400 random slides from the given set of 500 are trained, while the other 100 slides left out for validation." The version of the XGBoost used at this stage is not reported.

**Patient Level Stage Classification:** The description given by the authors for patient level stage classification is "predicting patient-level pN-stage is automatically determined by slide-level metastases predictions."

As given in the Camelyon 17 paper [7]

- pN0 is no ITC, micro or macro metastases found
- pN0(i+) is only ITC found
- pN1(mi) is micro metastases but no macro metastases found

- pN1 is metastases found in 1-3 slides at least one is a macro metastasis
- pN2 is metastases found in 4-9 slides at least one is a macro metastasis

The implementation applied these rules to the camelyon 17 labels of the five slides for each patient to determine the patient pN stage.

### 2.3 Liu algorithm

The third paper that was attempted for re-implementation was the paper by [8]. The re-implementation used the methods as detailed in the paper, it was not supplemented with additional code, as none has been released.

**System specification and software** The paper states that 8 NVIDIA Pascal GPUs were used for training and that the Tensorflow framework was used.

**Dataset splitting** This paper used the Camelyon 16 dataset and not the Camelyon 17 dataset. It is stated in table 2 of appendix A in [8] there were 215 slides in the training set and 54 in the validation set. It is not stated how slides were allocated to training and validation sets.

**Stain normalisation** No stain normalisation was used, it is stated in [8] that "Although the current leading approaches report improvements from colour normalisation, our experiments revealed no benefit"

**Tissue segmentation** The authors stated in the paper "To reduce computation, we removed background patches (gray value ¿ 0.8)". It is not stated at what level this tissue segmentation was carried out.

**Patch extraction** The details given by the authors for patch extraction are "For each input patch, we predict the label of the center 128 by 128 region". It is stated that the input size to the CNN is 299 by 299 pixels and so that must be the overall size of the patches. The paper of [8] describes several experiments with patches at different magnifications and combinations of different magnifications. The best results were found at 40x magnification in Camelyon 16 that corresponds to the level zero patches.

The authors describe their method for selecting patches to avoid bias as "Avoiding biases towards slides containing more patches (both normal and tumour) required careful sampling. First, we select "normal" or "tumour" with equal probability. Next, we select a slide that contains that class of patches uniformly at random, and sample patches from that slide." It does not describe how the patches are sampled from the slide. It is also stated that "we add jitter to the patch extraction process such that each patch has a small x,y offset of up to 8 pixels.", describing it as jitter may imply that the 128 by 128 patches are extracted on a regular grid but it is not clear if this is the case.

The exact number of patches used for training is not clear, the size of the training dataset is mentioned as "our large dataset size ($10^7$ patches)", in the appendix it gives the number of normal and tumour patches in total on all the slides as 13 million normal patches and 0.87 million tumor patches for the training set and 3.8 million normal and 0.28 million tumour for the validation set.

For labelling the patches the authors of the [8] paper state "We label a patch as tumour if at least one pixel in the center region is annotated as tumour."

**Patch augmentation** The paper of [8] includes extensive augmentation of the patches. The authors state that "we apply several data augmentations. First, we rotate the input patch by 4 multiples of 90◦, apply a left-right flip and repeat the rotations."

In addition to augmentation by flipping and rotating the authors state that extensive colour augmentations were carried out as followed, "Perturb colour: brightness with a maximum delta of 64/255, saturation with a maximum delta of 0.25, hue with a maximum delta of 0.04, and contrast with a maximum delta of 0.75."

**Patch classification** The authors state that "We utilize the Inception (V3) architecture with inputs sized 299 by 299". In addition giving the following details about the hyper parameters, "We trained our networks with stochastic gradient descent, with 8 replicas, batch size of 32 per replica. We used RMSProp with momentum of 0.9, decay of 0.9 and $\epsilon = 1.0$. The initial learning rate was 0.05, with a decay of 0.5 every 2 million examples."

**Hard negative mining** Hard negative mining is not mentioned in the paper.

**Heatmap generation** "We run inference across the slide in a sliding window with a stride of 128 to match the center region's size. For each patch, we apply the rotations and left-right flip to obtain predictions for each of the 8 orientations, and average the 8 predictions."

**Whole slide classification** The authors state that "For each slide, we report the maximum value in the heatmap as the slide-level tumour prediction"

**Lesion level classification** The authors of the paper [8] state that "we use a non-maxima suppression method that repeats two steps until no values in the heatmap remain above a threshold t: (1) report the maximum and corresponding coordinate, and (2) set all values within a radius r of the maximum to 0. Because we apply this procedure to the heatmap, r has units of 128 pixels. t controls the number of points reported and has no effect on the FROC unless the curve plateaus before 8 FP. To avoid erroneously dropping tumour predictions, we used a conservative threshold of t = 0.5." For values of r the paper stats "By contrast, our non-maxima suppression approach is relatively insensitive to r between 4 and 6".

**Patient level stage classification** Predictions are not made for the Camleyon 17 dataset so no patient level slide classification is carried out.

# 3 Reimplementation details

## 3.1 Common architecture

The hardware and structure of the software used for reimplementation was standardised across all the papers. The standard software framework used across all the papers was PyTorch which was selected for the familiarity of the team and perceived usability.

The standard framework split the training slides into a train and validation set of slides at the slide level. This ensured that all patches from a slide were either in the train or validation sets. It is assumed that adjacent patches will be very similar to each other and therefore keeping them in the same set would improve generalisation. The test set is defined in [9] no splitting is required as it is kept as a separate holdout set throughout the training process.

All reimplementations use a standardised patch labelling method based on downsampling the image, drawing the annotations at the downsampled scale, and using intersection with those annotations to dictate the label for each patch. This means that the labelled area must have a width and height in pixels that is a factor of two. Therefore to have patches of different sizes, they can either be cropped out of a larger labelled area (e.g. a 224 patch cropped out of a 256 area) or a border can be added to a smaller labelled central area (e.g. add 48 pixel border all around a 128 pixel labelled area to give a 224 patch). An alternative method to downsampling is to create an image at level zero containing the labels. This gives the flexibility of creating patches of any size without cropping from larger areas or adding borders to smaller areas. However, the labelled image at level zero will be very large and the whole dataset will therefore require large amounts of storage space. The processing of these labelled images at level zero is also computationally expensive. This is one area where the published papers lack information it is not clear which method is being used. As none of the papers detailed their methods for patch labelling, the first approach was applied consistently for all of the reimplementations in order to save space and computation time. If the papers applied other approaches this could be a source of variation in patch labels, particularly around the edges of annotations. The same concept also applies to the generation of heatmaps as in a heatmap one pixel corresponds to one patch.

The hardware used was an NVIDIA DGX-1 which was the best hardware available to this project. The structure of the project was standardised using the cookie cutter template with added docker support to help with reproducibility.

**System specification and software** The pipeline has been implemented on an NVIDIA DGX-1 with 2 Intel Xeon E5-2698 v4 CPUs and 8 SXM2 NVIDIA Tesla V100 GPUs. The core software packages used are:

- Python version 3.6.9
- PyTorch version 1.7.1
- PyTorch-lightning version 1.1.1

## 3.2 Wang algorithm

**Dataset splitting** The 270 slides were split into a training and validation set with 80% of the slides making the training set and 20% of the slides the validation set. The slides were sorted into each set based on the slide level label.

**Stain normalisation** Stain normalisation was not included as an efficient and working stain normalisation algorithm was not ready available.

**Tissue segmentation** The reimplementation was carried out on a thumbnail at level 5 of the image pyramid using the scikit-image package using the following steps:

- Convert the image from the RGB to HSV colour space
- Finding optimal threshold for each channel using Otsu's method
- Combining the mask images for the H and S channels

**Patch extraction** Patches of 256x256 pixels were extracted at level 0. The patches were created on regular grid with a stride of 32 pixels between each patch and so were overlapping. The patches were labelled using the following method:

1. Scale the Camelyon16 annotations from level 0 to level 5 by dividing their coordinates by 32 and rounding the nearest integer.
2. Create the labels image with the same width and height as the WSI at level 5, filled with zeros.
3. Draw the annotations onto the labels image using OpenCV's fillPoly function.
4. Apply the tissue segmentation algorithm to the WSI at level 5, and set any pixel that is not tissue to 0 in the labels image.
5. Apply a max pooling operation to the labels image, with a kernel size of 4 and a stride of 1, to create a label for each patch.

2,000,000 patches of each class were extracted for the train set and 500,000 patches of each class for valid. These were randomly sampled without replacement.

**Patch augmentation** In the reimplementation the following augmentations were applied using using the transforms package from PyTorch Vision version 0.8.2:

- Random rotation for one of four values: 0, 90, 180, or 270 degrees.
- Random crop down to 224x224.

**Patch classification** The patch classifier model and parameters used are as following:

- The network used for patch classification was GoogleNet from Torchvision models.
- The dropout for the final fully connected layer was set to 0.5.
- The network was initialised using the default random weights.
- The cross-entropy loss function from PyTorch was used.
- The SGD optimiser from PyTorch optim with weight $decay = 0.0005$ was used.
- The initial learning rate was set to 0.01 and it divided by 2 after every 50,000 batches.
- The model used 8 GPUS and distributed Data Parallelism (DDP).
- The maximum epochs was set to 3.
- The weights of the model used for inference are the weights after the epoch with the highest validation accuracy during training.
- The batch size was set to 32.

**Hard negative mining** After the initial classification was carried out, the patch classifier was run for every patch in the training set. All false positives were added to the training set and the classifier was retrained starting from the previous weights. This meant that 500,000 patches were added to the training set.

**Heatmap generation** In the reimplementation 256x256 non overlapping patches were created for each slide. Any patches that did not contain any tissue as determined by the tissue segmentation algorithm were given a probability of zero. The remaining patches were passed through the patch classifier to get a predicted probability for the tumor class. These probabilities were then assembled into a heatmap with each pixel representing a patch.

**Whole slide classification** Features were then generated from the heatmaps. In order to do this, the heatmap for each slide was thresholded at a series of different levels (0.5, 0.6, 0.7, 0.8 and 0.9) to produce a set of binary images. For each binary image, the following global features were computed:

- Ratio of tumour pixels to tissue pixels.
- Sum of all the probabilities in the tumour areas.

Connected-component analysis was applied to split the image into regions - thought to correspond to different lesions. This was done using the binary image generated by thresholding each heatmap at 0.5. Connect-component analysis was implemented using the ConnectedComponents class in scikit-image.

For the two largest regions, based on number of pixels, the following 10 features were extracted using the regionprops function from scikit-image.

- area of region
- the eccentricity of an ellipse that has the same second moments as the region
- the ratio of the area to the area of the bounding box
- the bounding box area
- the major axis length of the ellipse with the same second moments of area as the region
- the max intensity within the region
- the mean intensity within the region
- the min intensity within the region
- the aspect ratio of the bounding box
- the ratio of the area of the region to the area of the convex area

This gave a total of 30 features to use in the slide classification.

The reimplementation created a random forest classifier using the random forest classifier function from the scikit-learn ensemble package with the following parameters:

- number of estimators = 100
- bootstrapping was turned on
- maximum features was set to $'sqrt'$

**Lesion extraction** The reimplementation proceeded as follows

- Threshold the heatmaps from the initial model at 0.9
- Apply the connected components algorithm as implemented by the label function in scikit-image measure.
- extract the centre point using the regionprops function from scikit-image for each component
- sum the intensity values for that component on both the hard negative mined and initial model to give a lesion score

**Patient level stage classification** The paper uses only Camelyon 16 so does not report patient level classification.

## 3.3 Lee algorithm

**System specification and software** The pipeline has been implemented on an NVIDIA DGX-1 with 2 Intel Xeon E5-2698 v4 CPUs and 8 SXM2 NVIDIA Tesla V100 GPUs. The core software packages used are:

- Python version 3.6.9
- PyTorch version 1.7.1
- PyTorch-lightning version 1.1.1
- Torchvision version 0.8.2

**Dataset splitting** The datasets were split to train and validation sets at the slide level that is all patches from the same slide will be in the same dataset. In addition, the Camelyon 17 data set was split at the patient level to ensure that all the slide for the same patient will be either in train or in validation set. The Camelyon 17 data set contains both annotated and unannotated slides.

Tumour and normal slides were split separately so that 62% of the tumour slides and 62% of normal slides were selected for the train set. The slides were selected randomly. The 62% correspond to the percentage of the patches stated by the authors in paper [6]. In addition for Camelyon 17, it was ensured that 62% of the annotated slides were in the training set. Although the unannotated slides from Camelyon 17 have been included in dataset splitting stage but they won't be used for training the patch classifier, instead they will be used for slide level classification.

**Stain normalisation** No stain normalisation was applied, both due to the lack of details in the paper [6] and lack of access to any working preimplemented stain normalisation algorithm.

**Tissue segmentation** The segmentation is done at level 6. In the Camelyon whole slide images areas in which there is no data are saved as either pure white or pure black. In this method any pure black pixels on the slide were converted to pure white pixels. The image then was converted to grey scale using scikit-image RGB2GRAY. This resulted in all values of pixels on the slide to be between zero and one. Any pixel having value less than or equal 0.8 will be then considered as tissue.

**Patch extraction** Patches have been extracted at level zero on a regular grid with a stride of 256 pixels in both x , y directions from the whole slide images. The patch size is $256 \times 256$ pixels. Patches were randomly cropped using RandomCrop from transforms library in torchvision package to $240 \times 240$ pixel areas from the larger patches of size $256 \times 256$. From all the patches 47574 each of tumour patches and normal patches were randomly sampled without replacement for the training patch set and 29000 each of tumour patches and normal patches for the validation patch set. To determine the class of the patches, following procedures were carried out for each slide:

- A thumbnail of the slide where every pixel represents a patch of $256 \times 256$ pixels was created
- Tissue segmentation algorithm was applied to determine which pixels are tissue or background.
- The ground truth annotation masks contain the outline of polygons in level zero coordinates. Each coordinate is divided by 256 to give the coordinate as the same level as thumbnail. A blank mask with the same size as the thumbnail is created and the polygons plotted into this mask. Polygons are plotted using cv2.fillPoly. Each pixel covered by a polygon is then considered as a tumour patch.

**Patch augmentation** Generated patches were then normalised between zero and one. Normalize from transforms library in torchvision package has been used for this purpose.

**Patch classification** The patch classifier model and parameters used are as following:

- The networks used for patch classification was DenseNet-121 from torchvision models.
- Two fully connected layers were added at the end of DenseNet-121.
- The fully connected layers were implemented using the Linear function from pytorch. The first linear layer has 1000 neurons and second one has 2 neurons.
- The network used pretrained weights from torchvision.
- The cross-entropy loss function from pytorch was used.
- The SGD optimizer from pytorch optim with $momentum = 0.9$ and weight $decay = 0.0001$ was used.
- The initial learning rate was set to 0.1 and it divided by 10 after every 10 epochs.
- The model used 8 GPUS and distributed Data Parallelism (DDP).
- The maximum epochs was set to 15. Validation accuracy was monitored and the training stopped if the there was no increase in the validation accuracy for 5 epochs.
- The weights of the model used for inference are the weights after the epoch with the highest validation accuracy during training.
- The batch size was set to 64.

**Hard negative mining** This was implemented by predicting every patch on every training slide, finding all the false positives. Since the number of false positive patches were so high, the same number of patches as the original number of the normal patches in the training were added. The patches with the highest probabilities were used. The model was retrained on these new patches and the weights were initialised with the weight from the model before hard negative mining.

**Heatmap generation** In the reimplementation the $256 \times 256$ pixel patches from the thumbnails were considered as a single pixel in the heatmap.

**Whole slide classification** The following steps were carried out to extract the features from heatmaps:

- Threshold the heatmap to produce a binary image. Values in the $heatmap \geq 0.58$ are set to true else values are false.
- The rows and columns of true values in the binary image are recorded as coordinates.
- The DBScan algorithm from scikit-learn version (0.23.2) with eps of 3 and min samples of 20 is applied to the coordinate .
- This creates numbered clusters plus a set of outliers of single points with the same number.
- Renumber all the points in the outliers as clusters with different numbers.
- Create an image where each pixel is either labelled zero or with the number of the cluster.
- Use the region props function in scikitimage (0.17.2 ) to extract the following values for each cluster: area, major axis length, minor axis length, mean intensity, min intensity and max intensity, the density is calculated as 1 / area.
- Seven values are saved for the three clusters with the largest areas giving a total of 21 features to train the classifier.

The XGBClassifier function from xgboost(1.2.0) package with default parameters was used. Two separate classifiers were trained one for classifying Camelyon 16 slides into two classes, tumour and normal, and one for classifying Camelyon 17 slides into four classes, itc, micro, macro, negative.

**Patient Level Stage Classification** The rules outlined in the paper [7] were used for patient level classification.

## 3.4 Liu algorithm

**System specification and software** The pipeline has been implemented on an NVIDIA DGX-1 with 2 Intel Xeon E5-2698 v4 CPUs and 8 SXM2 NVIDIA Tesla V100 GPUs. The core software packages used are:

- Python version 3.6.9
- PyTorch version 1.7.1
- PyTorch-lightning version 1.1.1
- Torchvision version 0.8.2

**Dataset splitting** The datasets were split to train and validation sets at the slide level, that is all patches from the same slide will be in the same dataset. Tumour and normal slides were split separately so that 80% of the tumour slides and 80% of normal slides were selected for the train set. The slides were selected randomly. The 80% corresponds to the 215 training slides of the total of 269 Camelyon 16 slides stated by the authors in paper [8].

**Stain normalisation** No stain normalisation was applied.

**Tissue segmentation** The segmentation was done at level 6. In the Camelyon datasets whole slide images areas in which there is no data are saved as either pure white or pure black. In this method any pure black pixels on the slide were converted to pure white pixels. The image then was converted to grey scale using scikit-image RGB2GRAY. This resulted in all values of pixels on the slide to be between zero and one. Any pixel having value less than or equal 0.8 were then considered as tissue.

**Patch extraction** Patches have been extracted at level zero on a regular grid with patch size of $128 \times 128$ pixels and a stride of 128 pixels in both x and y directions from the whole slide images. The labels for the patches were calculated based on the $128 \times 128$ pixel patches. A border of the surrounding pixels was extracted to increase the total patch size to $307 \times 307$ pixels as a larger patch size was required as input for the network. The probability of the whole patch was considered to be only the probability for the central $128 \times 128$ pixels.

From all the patches 5,000,000 each of tumour patches and normal patches were randomly sampled with replacement. Sampling was weighted so that there was the same probability of selecting a patch from any slide. For the validation patch set 1,250,000 each of tumour patches and normal patches were sampled by the same method.

To determine the class of the patches, following procedures were carried out for each slide:

- A thumbnail of the slide at level 7 was created, where every pixel represents a patch of $128 \times 128$ pixels.
- Tissue segmentation algorithm was applied to determine which pixels are tissue or background.
- The ground truth annotation masks contain the outline of polygons in level zero coordinates. Each coordinate is divided by 128 to give the coordinate as the same level as thumbnail. A blank mask with the same size as the thumbnail is created and the polygons plotted into this mask. Polygons are plotted using cv2.fillPoly. Each pixel covered by a polygon is then considered as a tumour patch.

**Patch augmentation** The number of tumour patches available to sample from was augmented by applying 90, 180 and 270 rotations to both the original patch and the patch reflected about the centre line. This was achieved using the hflip and rotate functions in the torchvision functional transforms package. Patches were randomly cropped to $299 \times 299$ pixel areas from the larger patches of size $307 \times 307$. Color augmentations were applied using the ColorJitter function from the torchvision package with these parameters brightness=0.25, contrast=0.75, saturation=0.25, hue=0.04. Cropped patches were then normalised between zero and one. The RandomCrop and Normalize functions from the transforms library in the torchvision package were used.

**Patch classification** The patch classifier model and parameters used are as following:

- The network used for patch classification was Inception v3 from torchvision models with two input classes, without pretrained weights and with an auxilliary branch.
- The cross-entropy loss function from pytorch was used. The total loss was the loss from the main output branch plus 0.4 $\times$ the output from the auxilliary branch.
- The RMSProp optimizer from pytorch optim with $momentum = 0.9$, $weight\_decay = 0.0$, $alpha = 0.9$ $eps = 1.0$ was used.
- The initial learning rate was set to 0.05 and it divided by 2 after every 62,500 batches.
- The model was run on 8 GPUS using Distributed Data Parallelism (DDP).
- The maximum epochs was set to 15. Validation accuracy was monitored and the training stopped if the there was no increase in the validation accuracy for 5 epochs.
- The weights of the model used for inference are the weights after the epoch with the highest validation accuracy during training.
- The batch size was set to 32.

**Hard negative mining** Hard negative mining was not implemented

**Heatmap generation** In the reimplementation each patch was considered as a single pixel in the heatmap representing the central 128 $\times$ 128 pixels. When carrying out inference to create the heatmap each patch was reflected and rotated by 90, 180 and 270 giving 8 orientations in total. This was again achieved using the hflip and rotate functions in the torchivision functional transforms package. The probability of each of these orientations was predicted by the model and the final probability used in the heatmap was the mean of the 8 orientations.

**Whole slide classification** The maximum probability on the heatmap was the probability for the slide. Slides with a maximum probability greater than 0.998 were classed as tumour and others as normal.

**Lesion level classification** A list of separate lesions within the heatmap were found, recording the lesion centre and probability as follows:

- All probabilities are recorded in an array along with row and column position in pixels
- All probabilities less than 0.5 are set to zero
- Repeat the following steps until no probabilities greater than zero are left.

  Find the row and column position of the maximum probability, record this point as a lesion centre

  Calculate the distance in pixels of all other pixels to this point

  Find all pixels where the distance to the maximum probability is less than 6, set the probability of these pixels to zero.

**Patient level stage classification** Patient level classification was not carried out

## References


1. Wang D, Khosla A, Gargeya R, Irshad H, Beck AH. Deep learning for identifying metastatic breast cancer. arXiv preprint arXiv:160605718. 2016;.

2. Bejnordi BE, Veta M, Van Diest PJ, Van Ginneken B, Karssemeijer N, Litjens G, et al. Diagnostic assessment of deep learning algorithms for detection of lymph node metastases in women with breast cancer. Jama. 2017;318(22):2199–2210.

3. Bejnordi BE, Litjens G, Timofeeva N, Otte-Höller I, Homeyer A, Karssemeijer N, et al. Stain specific standardization of whole-slide histopathological images. IEEE transactions on medical imaging. 2015;35(2):404–415.

4. Szegedy C, Liu W, Jia Y, Sermanet P, Reed S, Anguelov D, et al. Going deeper with convolutions. In: Proceedings of the IEEE conference on computer vision and pattern recognition; 2015. p. 1–9.

5. Lee S, Oh S, Choi K, Kim SW. Automatic classification on patient-level breast cancer metastases. 2019;.

6. Lee S, Cho J, Kim SW. AUTOMATIC CLASSIFICATION ON PATIENT-LEVEL BREAST CANCER METASTASES; 2016. https://camelyon17.grand-challenge.org/evaluation/results/.

7. Bandi P, Geessink O, Manson Q, Van Dijk M, Balkenhol M, Hermsen M, et al. From detection of individual metastases to classification of lymph node status at the patient level: the CAMELYON17 challenge. IEEE transactions on medical imaging. 2018;38(2):550–560.

8. Liu Y, Gadepalli K, Norouzi M, Dahl GE, Kohlberger T, Boyko A, et al. Detecting cancer metastases on gigapixel pathology images. arXiv preprint arXiv:170302442. 2017;.

9. Camelyon16. Camelyon 16 Challenge; 2016. Available from: https://camelyon16.grand-challenge.org.
